# How can we improve the care of patients with schizophrenia in the real-world? A population-based cohort study of 456,003 patients

**DOI:** 10.1038/s41380-023-02154-4

**Published:** 2023-07-21

**Authors:** Guillaume Fond, Bruno Falissard, Philippe Nuss, Cedric Collin, Stephanie Duret, Marc Rabbani, Isabelle De Chefdebien, Isabelle Tonelli, Pierre Michel Llorca, Laurent Boyer

**Affiliations:** 1 Centre for Studies and Research on Health Services and Quality of Life (CEReSS), AP-HM, Aix-Marseille University, Marseille, France; 2 Universite Paris-Saclay, UVSQ, Inserm, Developmental Psychiatry, CESP, Villejuif, France; 3 AP-HP, Service de Psychiatrie et de Psychologie Médicale, Paris, France; 4 IQVIA Operations, Paris, France; 5 Lundbeck SAS, Puteaux, Paris, France; 6 Otsuka Pharmaceutical, Paris, France; 7 University of Clermont Auvergne, Clermont-Ferrand, France

**Keywords:** Psychology, Neuroscience

## Abstract

An important step to improve outcomes for patients with schizophrenia is to understand treatment patterns in routine practice. The aim of the current study was to describe the long-term management of patients with schizophrenia treated with antipsychotics (APs) in real-world practice. This population-based study included adults with schizophrenia and who had received ≥3 deliveries of an AP from 2012–2017, identified using a National Health Data System. Primary endpoints were real-life prescription patterns, patient characteristics, healthcare utilization, comorbidities and mortality. Of the 456,003 patients included, 96% received oral APs, 17.5% first-generation long-acting injectable APs (LAIs), and 16.1% second generation LAIs. Persistence rates at 24 months after treatment initiation were 23.9% (oral APs), 11.5% (first-generation LAIs) and 20.8% (second-generation LAIs). Median persistence of oral APs, first-generation LAIs and second-generation LAIs was 5.0, 3.3, and 6.1 months, respectively. Overall, 62.1% of patients were administered anxiolytics, 45.7% antidepressants and 28.5% anticonvulsants, these treatments being more frequently prescribed in women and patients aged ≥50 years. Dyslipidemia was the most frequent metabolic comorbidity (16.2%) but lipid monitoring was insufficient (median of one occasion). Metabolic comorbidities were more frequent in women. Standardized patient mortality remained consistently high between 2013 and 2015 (3.3–3.7 times higher than the general French population) with a loss of life expectancy of 17 years for men and 8 years for women. Cancer (20.2%) and cardiovascular diseases (17.2%) were the main causes of mortality, and suicide was responsible for 25.4% of deaths among 18–34-year-olds. These results highlight future priorities for care of schizophrenia patients. The global persistence of APs used in this population was low, whereas rates of psychiatric hospitalization remain high. More focus on specific populations is needed, such as patients aged >50 years to prevent metabolic disturbances and 18–34-year-olds to reduce suicide rates.

## Introduction

The World Health Organization reports that 20 million people worldwide have schizophrenia [1]. There is a need to improve the care of individuals with schizophrenia, but there is a paucity of recent objective real-world data required to aid the development of concrete strategies for advances in schizophrenia care.

There have been concerted efforts to shift care from the hospital to the outpatient setting to reduce the burden on patients, their relatives and on hospitals [2]. Therefore, it is important to know the proportion of patients with schizophrenia who require psychiatric hospitalization versus adequate ambulatory care. The absence or delay of antipsychotic drugs (APs) treatment and AP withdrawal are primary causes for psychiatric hospitalization [3–7]. Medication observance/compliance is a major issue in all chronic illnesses [8, 9] but is particularly problematic in schizophrenia for various reasons, including poor patient insight into their illness, mental stigma, treatment refusal, lack of effectiveness and adverse events with APs [10, 11]. Systematic reviews have suggested highly variable compliance to APs of 24–90% [12, 13]. Long-acting injectable APs (LAIs) have demonstrated treatment benefits over oral APs in clinical trials and real-world studies of patients with schizophrenia, including reducing relapse or hospitalization risk (by 8–56%) and fewer all-cause discontinuations and withdrawals due to adverse events or lack of efficacy [14–16].

Increased suicide risk and comorbid major depressive disorders (MDD) are common in schizophrenia and are frequent causes of hospitalizations [17]. Comorbid MDD (the most common cause of suicide) is present in nearly 50% of patients with schizophrenia, but is poorly screened and treated due to confusion with the negative symptoms of schizophrenia [18].

The long-term use of APs is associated with adverse events, including increased cardiometabolic risk through an unknown mechanism [19]. Cardiovascular disease and cancer are among the most significant drivers of mortality in patients with schizophrenia [20–23]. Lifestyle and genetic factors may contribute to the increased risk of cardiovascular mortality and there is also growing evidence of cardiometabolic disturbances early in schizophrenic disease [20, 24, 25]. People with schizophrenia may also receive sub-optimal support for metabolic conditions, which could worsen cardiovascular disease outcomes [26]. To address this, many strategies have been proposed, such as regular cardiometabolic follow-up of patients treated with APs and promoting lifestyle interventions [27, 28]. It is important to collect real-world data to assess the occurrence of cardiometabolic complications and to determine if the aforementioned strategies are providing an effect.

An important step to identifying unmet needs and treatment gaps, and to subsequently provide guidance on improving management and outcomes for patients with schizophrenia, is to understand treatment patterns and outcomes in routine clinical practice. We conducted a review of data from the French National Health Data System (Système National des Données de Santé; SNDS) to study pharmaceutical treatment of schizophrenia over a period of 6 years in real-world practice. The SNDS is composed of several databases that collect and collate homogenous information on diagnoses, medical claims, and cause of death for the majority of the population in France. The objectives of this analysis were to describe AP prescription patterns, healthcare utilization, metabolic disturbances and mortality in patients with schizophrenia.

## Methods

### Study design and data sources

This was an observational study of a cohort of patients with schizophrenia using data from the SNDS between 1st January 2012 and 31st December 2017 (timelines of study summarized in Fig. S1). The study adheres to the STrengthening the Reporting of OBservational studies in Epidemiology (STROBE) guidelines [29]. The STROBE checklist is shown in Table S1.

SNDS [30] consolidates data from multiple databases including: the French Health Insurance Database (SNIIRAM) [31]; the Programme for the Medicalisation of Information System (PMSI) [32]; and the Epidemiological Center on the Medical Causes of Death (Center d’épidémiologie sur les causes médicales de décès; CépiDc) [33].

SNIIRAM is a computerized system that collates individual anonymized data regarding all reimbursements for healthcare costs issued to members of one of the compulsory health insurance schemes, which constitutes approximately 99% of all French residents (~65 million individuals) [34]. It is composed of the Inter-scheme Consumption Data (Données de Consommation Inter-Régimes; DCIR), which records reimbursement and treatment data, and the registry of long-term diseases (LTD; Affection de Longue Durée; ALD), which lists the 30 chronic diseases which are exempt from payment.

PMSI is a French hospital discharge database that facilitates medical-economic analysis of information on the diagnosis and hospitalization of patients. PMSI is divided into medicine-surgery-obstetrics-dentistry, psychiatry, after care services and home hospital care. Only data from the medicine-surgery-obstetrics and psychiatry were used in this study.

CépiDc, which is a part of the French National Institute of Health and Medical Research (INSERM), produces annual statistics on the cause of death in French territories (both mainland and overseas, with the exception of the Department of Mayotte, Africa). Data are based on the death certificate, and this information is sent to INSERM, who records the causes of death. At the time of this analysis, CépiDc data were available for 2013–2015 in the SNDS, and so reported data on mortality are limited by data availability within this timescale.

### Patients

Eligible patients were identified in a multi-stage selection process (Fig. S2). In the initial selection stage, patients with schizophrenia were identified in the database from 2010 to 2017 (2 years before the dates for the second stage of the selection process to identify additional information on comorbidities and comedications in the period before inclusion in this analysis) based on the criteria of an active long-term disease during the study with an international classification of diseases—10th revision (ICD-10) diagnostic code of F2.X for schizophrenia (and related disorders) (Table S2) and/or at least one hospital stay in the medicine-surgery-obstetric sector with a main diagnosis or related diagnosis indicative of schizophrenia, and/or at least one hospital stay with a main diagnosis or associated diagnosis indicative of schizophrenia in psychiatry (classified as part-time, full-time or outpatient status).

In the second stage, in which AP users were identified, patients who had at least 3 deliveries (on different dates) of Anatomical Therapeutic Chemical class N05A AP agents (except lithium) between 2012 and 2017 were included (Table S3). Key exclusion criteria are detailed in Fig. S2.

### Study endpoints

The co-study endpoints measured prescription patterns, healthcare utilization, metabolic disturbances and mortality in patients with schizophrenia in France. Other endpoints such as the impact of switching from oral APs to LAIs will be reported elsewhere.

### Data extraction

#### Sociodemographic data

Data extracted from SNDS included patient characteristics on the index date, including age group (18–34 years, 35–49 years or ≥50 years [age categories were selected based on previously published data from the QUALIFY study [35] and lack of evidence in elderly patients]), sex and supplementary French Universal Health Cover (Couverture Maladie Universelle Complémentaire). The supplementary French Universal Health Cover is a complementary social security program concerning healthcare for patients whose income is below a set amount [36] and is awarded to people who are not covered under the more general Social Security Insurance (SHI; assurance maladie) system [37]. Receipt of supplementary French Universal Health Cover is a proxy for individuals with very low economic status [38–40].

#### Prescription patterns

AP treatments between 2012 and 2017, including details of the specific AP delivered, exposure and persistence, were collated according to the following subgroups: all APs, oral APs, first-generation LAIs and second-generation LAIs (Table S2). The measure of AP exposure was started as soon as an AP was reimbursed in the SNDS database. An AP exposure period was defined by a succession of AP deliveries, the delay between two deliveries of which does not mark an interruption, a stop or a treatment switch. Persistence to AP treatments was estimated by survival method in patients from exposure periods. AP persistence was defined as the time between starting and discontinuing an AP treatment without any gaps in deliveries. AP persistence is presented as median duration of exposure and as percentage of patients still exposed at 6, 12 and 24 months. The delivery of antidepressants, anxiolytics, anticonvulsants and opiate substitutes were also collated. Treatment patterns of different classes of APs (oral, first- and second-generation) were also assessed over time.

#### Healthcare utilization

Data were collected on healthcare utilization between 2012 and 2017, including psychiatric hospitalizations (part-time and full-time), related outpatient procedures (i.e., admission to, and care in, mental health outpatient center, part-time therapy center or other care settings), medical consultations with a psychiatrist, general practitioner or other specialists.

#### Metabolic disturbances

Comorbidities including dyslipidemia, diabetes and hypertensive disease were collated at inclusion and during the entire monitoring period. Comorbidities were identified at index date using algorithms including chronic disease status of patients (Affection de Longue Durée) (Table S3), specific drug dispensations used as proxy (Table S3), and details of hospitalizations for specific conditions. Biological metabolic monitoring procedures of lipid balance, glucose levels, urine tests and blood counts were collated. Cardiovascular medication deliveries were collated (Table S3).

#### Mortality

For every death, ICD-10 code, sex, date of birth, date of death, and cause of death were recorded on the certificate. If the information on the death certificate was ambiguous, the clinician could state more than one cause of death.

### Statistical analysis

No formal sample size estimation was necessary, but with approximately 86% of the adult French population included in the SNDS, a sample size of between 400,000 and 450,000 schizophrenia patients was expected. Based on the premise that summarizing data without drawing probability-based inferences is a key aspect of observational study objectives [41, 42], descriptive statistics were initially used to describe demographic and clinical characteristics, prescribing patterns, mortality rates and healthcare resource utilization, with results presented as means (standard deviation [SD]) and medians (95% confidence interval [CI]) for continuous variables and frequencies and percentages for categorical variables. AP persistence was assessed using log-rank methods, according to sex, age group and specific AP delivery.

The standardized mortality ratio was estimated as the ratio of deaths observed in the study population to those expected based on the mortality rates of the French population. Age- and sex-specific death rates per 1000 inhabitants in the French general population were obtained from national statistical data (INSEE) [43]. Age- and sex-standardized mortality rates were adjusted for differences in the age distribution of the population by applying the observed age- and sex-specific mortality rates for the study population to the French population distribution. Analyses were performed using the software SAS® Enterprise Guide v7.4 (SAS Institute North Carolina, USA).

## Results

### Patient characteristics

From the SNDS, 585,718 patients were identified who had at least one diagnosis of schizophrenia during the period 2010–2017. Of these, 585,458 (99.96%) patients had at least three deliveries of an AP, identified between 2012 and 2017. 456,003 of these patients were eligible for the current analysis because they were enrolled in the régime général (RG) or a local mutual association (section locale mutualiste (SLM)), and had validated sociodemographic data on the index date (Fig. S2).

There were slightly more men (*n* = 244,984; 53.7%) than women (211,019; 46.3%) in the sample and those ≥50 years of age were the largest age category (Table 1). Almost 1 in 5 patients with schizophrenia were of lower socio-economic status (i.e., beneficiaries of supplementary French Universal Health Cover [Couverture Maladie Universelle Complémentaire]) and this was most pronounced in younger age groups with almost 1 in 3 receiving supplementary French Universal Health Cover (Table 1).

**Table 1:** Sociodemographic characteristics, prescriptions patterns, healthcare utilization, and metabolic disturbances of 456 003 patients with schizophrenia between 2012 and 2017^a^.

	Total population (*N* = 456,003) n (%)^b^	Men(*N* = 244,984, 53.7%) n (%)^b^	Women (*N* = 211,019, 46.3%) n (%)^b^	18–34 years (*N* = 101,362, 22.2%) n (%)^b^	35–49 years (*N* = 152,833, 33.5%) n (%)^b^	50 years and over (*N* = 201,808, 44.3%) n (%)^b^
Sociodemographic data						
Sex						
Men	244,984 (53.7)	–	–	70,096 (69.2)	91,679 (60.0)	83,209 (41.2)
Women	211,019 (46.3)	–	–	31,266 (30.8)	61,154 (40.0)	118,599 (58.8)
Supplementary French Universal Health Cover						
Yes	84,864 (18.6)	50,814 (20.7)	34,050 (16.1)	33,561 (33.1)	34,419 (22.5)	16,884 (8.4)
No	371,139 (81.4)	194,170 (79.3)	176,969 (83.9)	67,801 (66.9)	118,414 (77.5)	184,924 (91.6)
Prescription patterns^c^						
Oral APs	437,743 (96.0)	234,300 (95.6)	203,443 (96.4)	98,612 (97.3)	146,831 (96.1)	192,300 (95.3)
1 G LAI	79,882 (17.5)	50,553 (20.6)	29,329 (13.9)	16,503 (16.3)	29,505 (19.3)	33,874 (16.8)
Haloperidol decanoate	48,921 (10.7)	30,806 (12.6)	18,115 (8.6)	10,799 (10.7)	18,408 (12.0)	19,714 (9.8)
Zuclopenthixol	18,815 (4.1)	12,593 (5.1)	6222 (2.9)	5067 (5.0)	7590 (5.0)	6158 (3.1)
Fluphenazine	8785 (1.9)	5730 (2.3)	3055 (1.4)	939 (0.9)	2598 (1.7)	5248 (2.6)
Pipotiazine	6867 (1.5)	4302 (1.8)	2565 (1.2)	669 (0.7)	2176 (1.4)	4022 (2.0)
Flupentixol	6687 (1.5)	4016 (1.6)	2671 (1.3)	1149 (1.1)	2588 (1.7)	2950 (1.5)
2 G LAI	73,638 (16.1)	46,349 (18.9)	27,289 (12.9)	29,410 (29.0)	27,257 (17.8)	16,971 (8.4)
Risperidone microspheres	43,311 (9.5)	26,989 (11.0)	16,322 (7.7)	15,422 (15.2)	16,283 (10.7)	11,606 (5.8)
Paliperidone palmitate 1 month	41,110 (9.0)	27,051 (11.0)	14,059 (6.7)	17,267 (17.0)	15,610 (10.2)	8233 (4.1)
Aripiprazole monohydrate	12,428 (2.7)	7433 (3.0)	4995 (2.4)	6363 (6.3)	4360 (2.9)	1705 (0.8)
Paliperidone palmitate 3 months	3018 (0.7)	2091 (0.9)	927 (0.4)	1237 (1.2)	1227 (0.8)	554 (0.3)
Other psychotropic drugs						
Antidepressants	208,489 (45.7)	97,562 (39.8)	110,927 (52.6)	36,759 (36.3)	71,025 (46.5)	100,705 (49.9)
Anxiolytics	283,402 (62.1)	142,284 (58.1)	141,118 (66.9)	56,152 (55.4)	99,152 (64.9)	128,098 (63.5)
Anticonvulsants	129,794 (28.5)	66,031 (27.0)	63,763 (30.2)	23,609 (23.3)	46,749 (30.6)	59,436 (29.5)
Opiate substitution therapy	11,725 (2.6)	9567 (3.9)	2158 (1.0)	3799 (3.7)	6841 (4.5)	1085 (0.5)
Healthcare utilization						
Psychiatric hospitalization	238,861 (52.4)	131,369 (53.6)	107,492 (50.9)	69,477 (68.5)	86,228 (56.4)	83,156 (41.2)
Related outpatient procedure	208,988 (45.8)	116,910 (47.7)	92,078 (43.6)	61,536 (60.7)	76,349 (50.0)	71,103 (35.2)
Psychiatrist	198,732 (43.6)	103,771 (42.4)	94,961 (45.0)	51,705 (51.0)	72,666 (47.5)	74,361 (36.8)
Median (IQR)^d^	20.0 (5.0–53.0)	18.0 (4.0–49.0)	22.0 (5.0–57.0)	16.0 (4.0–44.0)	23.0 (6.0–60.0)	20.0 (5.0–52.0)
General practitioner	441,192 (96.8)	235,450 (96.1)	205,742 (97.5)	97,056 (95.8)	148,277 (97.0)	195,859 (97.1)
Median (IQR)^d^	25.0 (11.0–48.0)	21.0 (9.0–42.0)	30.0 (15.0–55.0)	16.0 (7.0–32.0)	25.0 (11.0–47.0)	31.0 (16.0–56.0)
Other specialists’ consultations	444,433 (97.5)	237,539 (97.0)	206,894 (98.0)	98,208 (96.9)	149,301 (97.7)	196,924 (97.6)
Metabolic disturbances						
Dyslipidemia	73,979 (16.2)	35,286 (14.4)	38,693 (18.3)	2104 (2.1)	15,706 (10.3)	56,169 (27.8)
Diabetes	43,052 (9.4)	18,883 (7.7)	24,169 (11.5)	1379 (1.4)	8275 (5.4)	33,398 (16.5)
Hypertensive disease	16,139 (3.5)	6356 (2.6)	9783 (4.6)	335 (0.3)	1682 (1.1)	14,122 (7.0)
Cardiovascular medications	109,645 (24.0)	44,360 (18.1)	65,285 (30.9)	5053 (5.0)	19,497 (12.8)	85,095 (42.2)

*AP* antipsychotic, *1* *G LAI* first-generation long-acting injectable antipsychotics, *2* *G LAI* second-generation long-acting injectable antipsychotics, *IQR* interquartile range.

^a^Data for sociodemographic characteristics, other psychotropic drugs and metabolic disturbances at the index date based on the 2-year historical period, while data on prescription patterns and healthcare utilization are based on the study period.

^b^Unless otherwise stated in left-hand column.

^c^*n* = 455,997. For AP drug prescription, 6 patients were excluded after the index date due to a change in their social situation (membership scheme not identified and no reimbursement found for medical treatments).

^d^Per patient over the course of the study period.

### Prescription patterns

Patients were followed-up for a mean (SD) of 61 (18) months and, during this period, 96% of patients received oral APs, 17.5% first-generation LAIs, and 16.1% second-generation LAIs (Table 1). Mean (SD) exposure to APs was 38.7 (23.2) months over the entire study period. Women received LAIs less frequently than men, and younger patients received second-generation LAIs more frequently than older patients (Table 1). The more frequently prescribed LAIs were haloperidol decanoate (10.7%), risperidone microspheres (9.5%), paliperidone palmitate 1 monthly (9.0%), zuclopenthixol (4.1%) and aripiprazole monohydrate (2.7%) (Table 1).

**Fig. 1:**
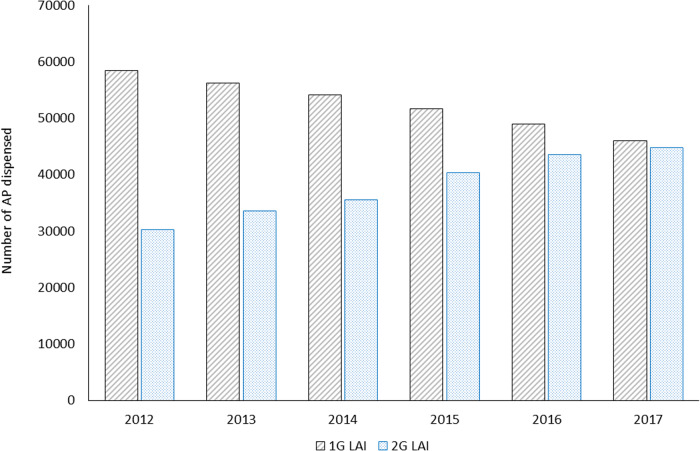
Long-acting injectable antipsychotic drugs dispensed in France from 2012 to 2017. *AP antipsychotic. 1G LAI, first-generation long-acting injectable antipsychotics. 2G LAI, second-generation long-acting injectable antipsychotic.

Median persistence of all APs, oral APs, first-generation LAIs and second-generation LAIs was, respectively, 5.4 months (95% CI: 5.4, 5.4), 5.0 months (95% CI: 5.0, 5.0), 3.3 months (95% CI: 3.2, 3.3), and 6.1 months (95% CI: 6.0, 6.1). Persistence rates at 24 months after initiation of treatment were 23.9% for oral APs, 11.5% for first-generation LAIs and 20.8% for second-generation LAIs. Persistence rates at 24 months were slightly higher for men than women for oral APs (25.1% and 22.4%, respectively), first-generation LAIs (11.8% and 11.1%, respectively), and second-generation LAIs (21.2% and 20.0%, respectively). The youngest age group (18–34 years of age) had the lowest persistence rate with all classes of APs (19.9%, 9.3% and 17.8% for oral APs, first-generation LAIs and second-generation LAIs, respectively) compared with the 35–49 years or ≥50 years age groups (26.1%, 12.4% and 22.8%, and 24.3%, 11.6% and 23.0%, respectively). Generally, there was a trend suggesting that second-generation LAIs were gradually increasing in use between 2012 and 2017 while first-generation LAIs were decreasing (Fig. 1).

Among other psychotropic drugs, 62.1% of the patients had at least one delivery of anxiolytics, 45.7% antidepressants and 28.5% anticonvulsants during the study period. Women were more frequently administered these treatments than men: anxiolytics were administered in 66.9% and 58.1%, antidepressants in 52.6% and 39.8%, and mood stabilizers in 30.2% and 27.0%, respectively. There were also differences in the frequency of these treatments according to age group, with a general trend towards an increase in the numbers of patients requiring treatment with age.

### Healthcare resource utilization

During the time-period of this study, 52.4% of patients with schizophrenia were hospitalized in psychiatric departments (Table 1); 49.2% were hospitalized full-time and 16.8% were hospitalized part-time for psychiatric-related issues. A total of 229,951 (50.4%) patients were hospitalized in medicine-surgery-obstetrics departments. Less than 50% of patients received treatment from an outpatient psychiatrist. There were no gender differences in healthcare resource utilization (Table 1).

### Metabolic disturbances

Overall, 16.2% of patients were diagnosed with dyslipidemia, 9.4% with diabetes, 3.5% with hypertension (Table 1). These rates were higher in women than in men (Table 1). Likewise, the use of cardiovascular medications was more frequent in women than in men (Table 1). Over the course of the study, blood glucose levels were monitored on a median of four occasions, but lipids were measured on a median of one occasion only.

### Mortality

Follow-up data between 2013 and 2015 from CépiDc were available for 438,024 patients, where 16,652 deaths were reported (4741, 5582 and 6329 occurred in 2013, 2014 and 2015, respectively). The standardized mortality rate per 1000 patients in 2013, 2014 and 2015, when compared with the general French population, was 27.0 versus 8.1, 30.9 versus 8.5 and 33.7 versus 9.0, respectively. The ratio of mortality rates was similar across the 3 years (3.3 in 2013; 3.6 in 2014 and 3.7 in 2015). The main causes of death were cancer (20.2%) and cardiovascular disease (17.2%; Table 2). Suicide was responsible for 5.9% of all deaths and mental and behavioral disorders for 9.1% (Table 2). The proportion of patients committing suicide was 25.4% among 18–34-year-olds, which was the most frequent cause of death in this age group (Table 2).

**Table 2:** Analysis of mortality in patients with schizophrenia receiving AP therapy between 2013 and 2015.

	Total population (*N* = 16,652)	Men (*N* = 8160)	Women (*N* = 8492)	18–34 years (*N* = 702)	35–49 years (*N* = 2437)	50 years and over (*N* = 13,513)
Sex						
Men	8160 (49.0%)	–	–	548 (78.1%)	1654 (67.9%)	5958 (44.1%)
Women	8492 (51.0%)	–	–	154 (21.9%)	783 (32.1%)	7555 (55.9%)
Median (Q1-Q3) age at death	69.0 (55.0–82.0)	62.0 (50.0–75.0)	77.0 (61.0–86.0)	–	–	–
Beneficiary of additional French Universal Health Cover	1491 (9.0%)	949 (11.6%)	542 (6.4%)	244 (34.8%)	540 (22.2%)	707 (5.2%)
Main causes of death						
Cancer	3360 (20.2%)	1730 (21.2%)	1630 (19.2%)	27 (3.8%)	399 (16.4%)	2934 (21.7%)
Cardiovascular disease	2859 (17.2%)	1268 (15.5%)	1591 (18.7%)	40 (5.7%)	250 (10.3%)	2569 (19.0%)
Mental and behavioral disorders	742 (9.1%)	742 (9.1%)	780 (9.2%)	62 (8.8%)	254 (10.4%)	1206 (8.9%)
Respiratory disorders	1374 (8.3%)	627 (7.7%)	747 (8.8%)	11 (1.6%)	79 (3.2%)	1284 (9.5%)
Other external causes of accidental injury	1144 (6.9%)	590 (7.2%)	554 (6.5%)	91 (13.0%)	244 (10.0%)	809 (6.0%)
Nervous system disorders	980 (5.9%)	454 (5.6%)	526 (6.2%)	18 (2.6%)	79 (3.2%)	883 (6.5%)
Suicide	800 (4.8%)	533 (6.5%)	267 (3.1%)	178 (25.4%)	336 (13.8%)	286 (2.1%)
Endocrine, nutritional and metabolic disorders	756 (4.5%)	297 (3.6%)	459 (5.4%)	9 (1.3%)	79 (3.2%)	668 (4.9%)
Gastrointestinal disorders	701 (4.2%)	371 (4.5%)	330 (3.9%)	23 (3.3%)	109 (4.5%)	569 (4.2%)
Main methods of suicide						
Drug overdose	195 (24.4%)	104 (19.5%)	91 (34.1%)	30 (16.9%)	97 (28.9%)	68 (23.8%)
Self-harming by hanging, strangulation, suffocation	348 (43.5%)	271 (50.8%)	77 (28.8%)	92 (51.7%)	144 (42.9%)	112 (39.2%)
Self harming by jumping into the void	141 (17.6%)	82 (15.4%)	59 (22.1%)	36 (20.2%)	57 (17.0%)	48 (16.8%)

## Discussion

This is, to our knowledge, one of the largest and most comprehensive real-world assessments of patients with schizophrenia treated with APs, conducted in a homogeneous national health system over a period of six years, with individuals derived from a broad spectrum of care settings (hospital and outpatient).

Our results suggest that during the studied period, more than half of the patients with schizophrenia were referred to full-time or day-time hospitalization, highlighting how complex this illness is to manage in outpatient settings. Furthermore, over half of the patients were not seen in outpatient psychiatric settings. Strikingly, while almost all patients had at least one contact with a general practitioner in the study period, less than half had contact with a psychiatrist. Schizophrenia spectrum disorders require a specialist follow-up, and these findings may illustrate patients’ reluctance to consult psychiatrists, due to forced hospitalizations and resulting post-traumatic consequences [44]. Measuring and improving patient-reported experience of psychiatric care is needed to increase compliance to psychiatric follow-up [45, 46]. Hospitalization was much more frequent in 18–34-year-old patients, who have more substance addiction-related comorbidities and lower insight into their illness [47]. This population should be targeted as a priority to improve compliance to psychiatric care. This is the aim of developing initiatives and programs targeting first episode psychosis in many countries, including France, which has more than 30 regional early intervention programs in operation or underway for 15–35-year-old patients [48, 49]. These programs also include suicide prevention, which is of note given that suicide represented the primary cause of mortality among 18–34-year-old patients in our study.

The first striking result is the very low AP persistence, but these values should be interpreted with caution. Oral APs and first-generation LAIs were first marketed long before the study period, whereas some of the second-generation LAIs were launched during the study period (2012–2017). Therefore, second-generation LAI persistence may be underestimated due to the recent addition of these drugs to the market. Over the study period, patients taking first-generation LAIs may have been switched to second-generation LAIs, and our results do show a trend for increasing second-generation and decreasing first-generation LAIs. However, consistent with previous studies, second-generation LAIs appear to have better persistence than oral APs [50–52]. This study should be replicated in 5 years, when more data for second generation antipsychotics will be available and there will be longer-acting formulations, which are expected to have a better persistence due to reduced injection frequency [53–55].

Considering the very high hospitalization rates in the 18–34 year olds (>68%), it remains to be discussed what the optimal LAI prescription rate should be in this population. Second-generation LAI prescriptions are much higher in this younger population (29%), possibly suggesting that prescribers use LAIs to prevent psychiatric hospitalizations specifically in this population. This may also reflect the recommendation to use second-generation LAIs that were published in 2013 (i.e., during the period of this study) [56]. However, most patients refuse LAIs for multiple reasons [57]. Current recommendations also encourage shared-decision making to increase adherence to treatment [57].

This study also reveals that a considerable proportion of patients who are taking APs also get prescribed antidepressant and/or anxiolytic drugs and/or anticonvulsants. This confirms the complexity of the management of schizophrenia, and emphasizes the importance of adequate screening and monitoring of comorbid symptoms [58]. Such prescriptions were observed to be more frequent in women, contrary to results published in a cohort of younger patients with schizophrenia (mean age 32 years; 74% men) [18]. This discrepancy is therefore probably due to greater representation of older women in the present cohort. Given the known association between anxiety or depression and cardiovascular disease [59, 60], the increased rates of these psychiatric conditions in this study are also explained by higher rates of cardiovascular comorbidities among women in our study. There was a high proportion of patients receiving cardiovascular drugs in this population (higher than the French national average of 5.2% of the population receiving pharmacological treatment for diabetes or 12.5% under 60 years of age receiving lipid-lowering drugs in 2019 [61, 62]) and as expected, many comorbidities, especially cardiometabolic conditions, were more frequent in older age-groups and in women. Moreover, cardiometabolic complications appeared early in a proportion of this population; 10% of the 35–49-year-old patients were diagnosed with dyslipidemia and 12% were prescribed cardiovascular drugs. It is also possible that cardiometabolic conditions are underestimated using the SNDS data, and indeed these conditions are generally under-diagnosed in patients with schizophrenia [63]. The rate of dyslipidemia may also be underestimated as patients had lipid assessments a median of only once during the whole study period of 6 years. However, this is in line with the recommendations of the French Drug Safety Agency (ANSM), i.e., one lipid test every 5 years on maintenance [64]. These results suggest that this frequency could potentially be shortened.

Strategies are clearly needed to prevent cardiometabolic dysfunction in patients with schizophrenia, which contributes to high mortality rates and is predictive of relapse [20, 65]. Lifestyle interventions may be effective, but pose a barrier to patients with motivational deficits [66, 67]. As different APs have different effects on weight gain [68–70] and metabolic function [71], another strategy to tackle cardiometabolic dysfunction is to seek alternative treatments that lack metabolic adverse events. However, updated international guidelines to help psychiatrists and general practitioners manage metabolic disturbances in schizophrenia are needed.

The higher standardized mortality rates for patients with schizophrenia than for the general population in France is consistent with data from other countries [17, 72, 73]. Schizophrenia was associated with a loss of life expectancy of around 17 years for men and 8 years for women compared with French national data from Institut National de la Statistique et des Etudes Economiques (INSEE) [43]. The finding that cancer and cardiovascular disease were the most frequent causes of death among older patients is in agreement with evidence supporting a significant association between schizophrenia and both cancer and cardiovascular mortality [74]. The same pattern of occurrence has been observed in other countries, including the USA and Sweden [72, 75]. In the current analysis, deaths in patients with schizophrenia were 3.3 to 3.7 times higher than in the general French population [17]. These data further highlight the need to tackle comorbidities among patients with schizophrenia.

Almost 1 in 5 patients in this study were deemed to be of very low socio-economic status, as they required supplementary French Universal Health Cover (compared with 9% of the general population [76]), and this reflects the difficulty experienced by patients in finding employment [77, 78]. This should alert public authorities to the need for implementation of procedures and collective strategies aimed at promoting recovery and healthy attitudes in this population [79]. Such patients are often under-represented in observational studies, which is a strength of the current analysis.

Study limitations are inherent to an observational study design and specific limitations in relation to this analysis include: as the source of the data was a claims database, it is not possible to determine if APs were administered optimally; diagnosis was based on hospital data and therefore some individuals may have been misclassified due to the lack of a hospital-based diagnosis [80]; because long-acting olanzapine injections are reserved for hospital delivery, information on this agent was not included; and similarly, other medications dispensed during a full-time hospital stay were not recorded. Future analyses could also extract more detailed data on the use of mood stabilizers such as lamotrigine, which is recommended when there is an insufficient response to APs. Likewise, the impact of antidepressants on outcomes would be interesting to assess, and because clozapine is frequently prescribed for more severe schizophrenia, then a separate analysis of clozapine use could be useful. Further analyses could also encompass mortality by cancer type in order to examine the potential association between APs and lung cancer attributed to smoking, for example, given the reported differential effects of first- and second-generation APs on smoking behavior [81]. The use of CMU-C as a proxy measure of low socioeconomic status may be considered an indirect approach but has demonstrated utility in studies evaluating the French population [38–40]. Other limitations stemming from the retrospective and claims-based design include the potential for missing data and cases that are not enrolled in the French healthcare system. Notably, our identified sample population of 585,718 with at least one diagnosis of schizophrenia and 456,003 of these patients meeting eligibility criteria was slightly larger than planned and likely reflects a higher than estimated prevalence of patients with schizophrenia and/or a greater proportion seeking care within the healthcare system. Nevertheless, our larger sample population falls within the reported range of 400,000 to 600,000 affected by schizophrenia in France [82], thus supporting the validity and sensitivity of our multi-step selection process and providing increased power for both descriptive and inferential interpretation of the obtained results.

## Conclusions

These results provide a roadmap of the priorities in the care of schizophrenia for the coming years. APs alone seem to be insufficient to manage schizophrenia in most cases. AP persistence remains low and rates of psychiatric hospitalization high. Women and patients aged >50 years appear particularly vulnerable to depressive/anxiety disorders and metabolic disturbances beginning as early as 35 years of age. Current health authority recommendations for screening lipid disturbances appear insufficient to prevent metabolic disturbances and their consequences, including cancer and cardiovascular diseases. Suicide prevention remains a priority in 18–34-year-old patients.

### Supplementary information

Supplementary materials
