# Supplementary material for: How can we improve the care of patients with schizophrenia in the real-world? A population-based cohort study of 456,003 patients
Source: Mol Psychiatry. 2023 Jul 21;28(12):5328–36. doi: 10.1038/s41380-023-02154-4 (PMC11041650; doi:10.1038/s41380-023-02154-4)
Supplement: Supplementary file 1 — Supplementary materials [file 41380_2023_2154_MOESM1_ESM.docx]

**Supplementary materials**

Table S1. STROBE checklist

|  | Item No | Recommendation | Page  No |
| --- | --- | --- | --- |
| **Title and abstract** | 1 | (*a*) Indicate the study’s design with a commonly used term in the title or the abstract | 1,2 |
|  |  | (*b*) Provide in the abstract an informative and balanced summary of what was done and what was found | 2 |
| Introduction | | | |
| Background/rationale | 2 | Explain the scientific background and rationale for the investigation being reported | 3,4 |
| Objectives | 3 | State specific objectives, including any prespecified hypotheses | 4 |
| Methods | | | |
| Study design | 4 | Present key elements of study design early in the paper | 4-8 |
| Setting | 5 | Describe the setting, locations, and relevant dates, including periods of recruitment, exposure, follow-up, and data collection | 4,5 |
| Participants | 6 | (*a*) *Cohort study*—Give the eligibility criteria, and the sources and methods of selection of participants. Describe methods of follow-up  *Case-control study*—Give the eligibility criteria, and the sources and methods of case ascertainment and control selection. Give the rationale for the choice of cases and controls  *Cross-sectional study*—Give the eligibility criteria, and the sources and methods of selection of participants | 5-6 |
|  |  | (*b*) *Cohort study*—For matched studies, give matching criteria and number of exposed and unexposed  *Case-control study*—For matched studies, give matching criteria and the number of controls per case |  |
| Variables | 7 | Clearly define all outcomes, exposures, predictors, potential confounders, and effect modifiers. Give diagnostic criteria, if applicable | 6 |
| Data sources/ measurement | 8* | For each variable of interest, give sources of data and details of methods of assessment (measurement). Describe comparability of assessment methods if there is more than one group | 6-8 |
| Bias | 9 | Describe any efforts to address potential sources of bias | NA |
| Study size | 10 | Explain how the study size was arrived at | 8 |
| Quantitative variables | 11 | Explain how quantitative variables were handled in the analyses. If applicable, describe which groupings were chosen and why | 8 |
| Statistical methods | 12 | (*a*) Describe all statistical methods, including those used to control for confounding | 8 |
|  |  | (*b*) Describe any methods used to examine subgroups and interactions | 8 |
|  |  | (*c*) Explain how missing data were addressed | NA |
|  |  | (*d*) *Cohort study*—If applicable, explain how loss to follow-up was addressed  *Case-control study*—If applicable, explain how matching of cases and controls was addressed  *Cross-sectional study*—If applicable, describe analytical methods taking account of sampling strategy | NA |
|  |  | (*e*) Describe any sensitivity analyses | NA |

| Results | | | |
| --- | --- | --- | --- |
| Participants | 13* | (a) Report numbers of individuals at each stage of study—eg numbers potentially eligible, examined for eligibility, confirmed eligible, included in the study, completing follow-up, and analysed | 9 |
|  |  | (b) Give reasons for non-participation at each stage | 9 |
|  |  | (c) Consider use of a flow diagram | Figure S2 |
| Descriptive data | 14* | (a) Give characteristics of study participants (eg demographic, clinical, social) and information on exposures and potential confounders | 9, Table 1 |
|  |  | (b) Indicate number of participants with missing data for each variable of interest | NA |
|  |  | (c) *Cohort study*—Summarise follow-up time (eg, average and total amount) | 9 |
| Outcome data | 15* | *Cohort study*—Report numbers of outcome events or summary measures over time | 9-11 |
|  |  | *Case-control study—*Report numbers in each exposure category, or summary measures of exposure |  |
|  |  | *Cross-sectional study—*Report numbers of outcome events or summary measures |  |
| Main results | 16 | (*a*) Give unadjusted estimates and, if applicable, confounder-adjusted estimates and their precision (eg, 95% confidence interval). Make clear which confounders were adjusted for and why they were included | NA |
|  |  | (*b*) Report category boundaries when continuous variables were categorized | 9 |
|  |  | (*c*) If relevant, consider translating estimates of relative risk into absolute risk for a meaningful time period | NA |
| Other analyses | 17 | Report other analyses done—eg analyses of subgroups and interactions, and sensitivity analyses | 9-11 |
| Discussion | | | |
| Key results | 18 | Summarise key results with reference to study objectives | 11-12 |
| Limitations | 19 | Discuss limitations of the study, taking into account sources of potential bias or imprecision. Discuss both direction and magnitude of any potential bias | 14-15 |
| Interpretation | 20 | Give a cautious overall interpretation of results considering objectives, limitations, multiplicity of analyses, results from similar studies, and other relevant evidence | 11-15 |
| Generalisability | 21 | Discuss the generalisability (external validity) of the study results | 11-15 |
| Other information | | | |
| Funding | 22 | Give the source of funding and the role of the funders for the present study and, if applicable, for the original study on which the present article is based | 16 |

*Give information separately for cases and controls in case-control studies and, if applicable, for exposed and unexposed groups in cohort and cross-sectional studies.

**Note:** An Explanation and Elaboration article discusses each checklist item and gives methodological background and published examples of transparent reporting. The STROBE checklist is best used in conjunction with this article (freely available on the Web sites of PLoS Medicine at http://www.plosmedicine.org/, Annals of Internal Medicine at http://www.annals.org/, and Epidemiology at http://www.epidem.com/). Information on the STROBE Initiative is available at www.strobe-statement.org.

Table S2. ICD-10 diagnostic codes for schizophrenia

|  | **Code**  **ICD-10** | **Description** |
| --- | --- | --- |
| Schizophrenia | F20.0 | Paranoid schizophrenia |
|  | F20.1 | Hebephrenic schizophrenia |
|  | F20.2 | Catatonic schizophrenia |
|  | F20.3 | Undifferentiated schizophrenia |
|  | F20.4 | Post-schizophrenic depression |
|  | F20.5 | Residual schizophrenia |
|  | F20.6 | Simple schizophrenia |
|  | F20.8 | Other schizophrenia |
|  | F20.9 | Schizophrenia, unspecified |
|  | F20 | Schizophrenia |
|  | F21 | Schizotypal disorder |
|  | F22 | Persistent delusional disorders |
|  | F23 | Acute and transient psychotic disorders |
|  | F24 | Induced delusional disorder |
|  | F25 | Schizoaffective disorders |
|  | F28 | Other nonorganic psychotic disorders |
|  | F29 | Unspecified nonorganic psychosis |

ICD-10, International Classification of Diseases – 10^th^ revision.

Table S3. Summary of drugs used to identify patients with schizophrenia

| **ATC class** | **INN** | **Generation of AP** | **LAI (Y/N)** |
| --- | --- | --- | --- |
| N05AA01 | Chlorpromazine | 1 | N |
| N05AA02 | Levomepromazine | 1 | N |
| N05AA06 | Cyamemazine | 1 | N |
| N05AA07 | Chlorproethazine | 1 | N |
| N05AB02 | Fluphenazine | 1 | Y |
| N05AB03 | Perphenazine | 1 | N |
| N05AB06 | Trifluoperazine | 1 | N |
| N05AB08 | Thioproperazine | 1 | N |
| N05AC01 | Periciazine | 1 | N |
| N05AC02 | Thioridazine | 1 | N |
| N05AC04 | Pipotiazine | 1 | Y |
| N05AD01 | Haloperidol | 1 | Y |
| N05AD02 | Trifluperidol | 1 | N |
| N05AD05 | Pipamperone | 1 | N |
| N05AD08 | Droperidol | 1 | N |
| N05AF01 | Flupentixol | 1 | Y |
| N05AF05 | Zuclopenthixol | 1 | Y |
| N05AG02 | Pimozide | 1 | N |
| N05AG03 | Penfluridol | 1 | N |
| N05AH01 | Loxapine | 1 | N |
| N05AH02 | Clozapine | 1 | N |
| N05AH03 | Olanzapine | 2 | N |
| N05AH04 | Quetiapine | 1 | N |
| N05AL01 | Sulpiride | 1 | N |
| N05AL03 | Tiapride | 1 | N |
| N05AL05 | Amisulpride | 1 | N |
| N05AL06 | Veralipride | 1 | N |
| N05AX08 | Risperidone | 2 | Y |
| N05AX12 | Aripiprazole | 2 | Y |
| N05AX13 | Paliperidone | 2 | Y |

ATC, Anatomical, Therapeutic and Chemical; INN, International Non-proprietary Name

Table S4: ICD-10 codes and drug codes identifying the comorbidities of interest

| **ICD-10** | **Description** |  |
| --- | --- | --- |
| ***Disorders of lipoprotein metabolism and other lipidemias*** | |  |
| E78 | Disorders of lipoprotein metabolism and other lipidemias |  |
| ***Diabetes*** |  |  |
| E10 | Diabetes mellitus type 1 |  |
| E11 | Diabetes mellitus type 2 |  |
| E12 | Malnutrition-related diabetes mellitus |  |
| E13 | Other types of diabetes mellitus, specified |  |
| E14 | Diabetes mellitus, unspecified |  |
| O24 | Diabetes mellitus during pregnancy |  |
| ***Hypertensive diseases*** | |  |
| G932 | Benign intracranial hypertension |  |
| H350 | Other retinopathies and retinal vascular disorders |  |
| H400 | Borderline glaucoma |  |
| I10 | Essential (primary) hypertension |  |
| I11 | Hypertensive heart disease |  |
| I12 | Hypertensive nephropathy |  |
| I13 | Hypertensive cardiac nephropathy |  |
| I15 | Secondary hypertension |  |
| I674 | Hypertensive encephalopathy |  |
| I70 | Atherosclerosis |  |
| K766 | Portal hypertension |  |
| R030 | Detection of increased blood pressure, without hypertension diagnosis |  |
| Z136 | Special test for screening for cardiovascular disorders |  |
| **ATC class** | **Description** |  |
| **Cardiovascular drugs** | |  |
| C02 | Antihypertensive |  |
| C03 | Diuretics |  |
| C07 | β-blockers |  |
| C08 | Calcium channel blockers |  |
| C09 | Agents acting on the renin-angiotensin system |  |
| **Dyslipidemias** | |  |
| C10 | Lipid-lowering agents |  |
| **Diabetes** | |  |
| A10 | Drugs used for diabetes |  |

ATC, Anatomical, Therapeutic and Chemical. ICD-10, International Classification of Diseases – 10^th^ revision.

Figure S1. Overview of data collection timelines


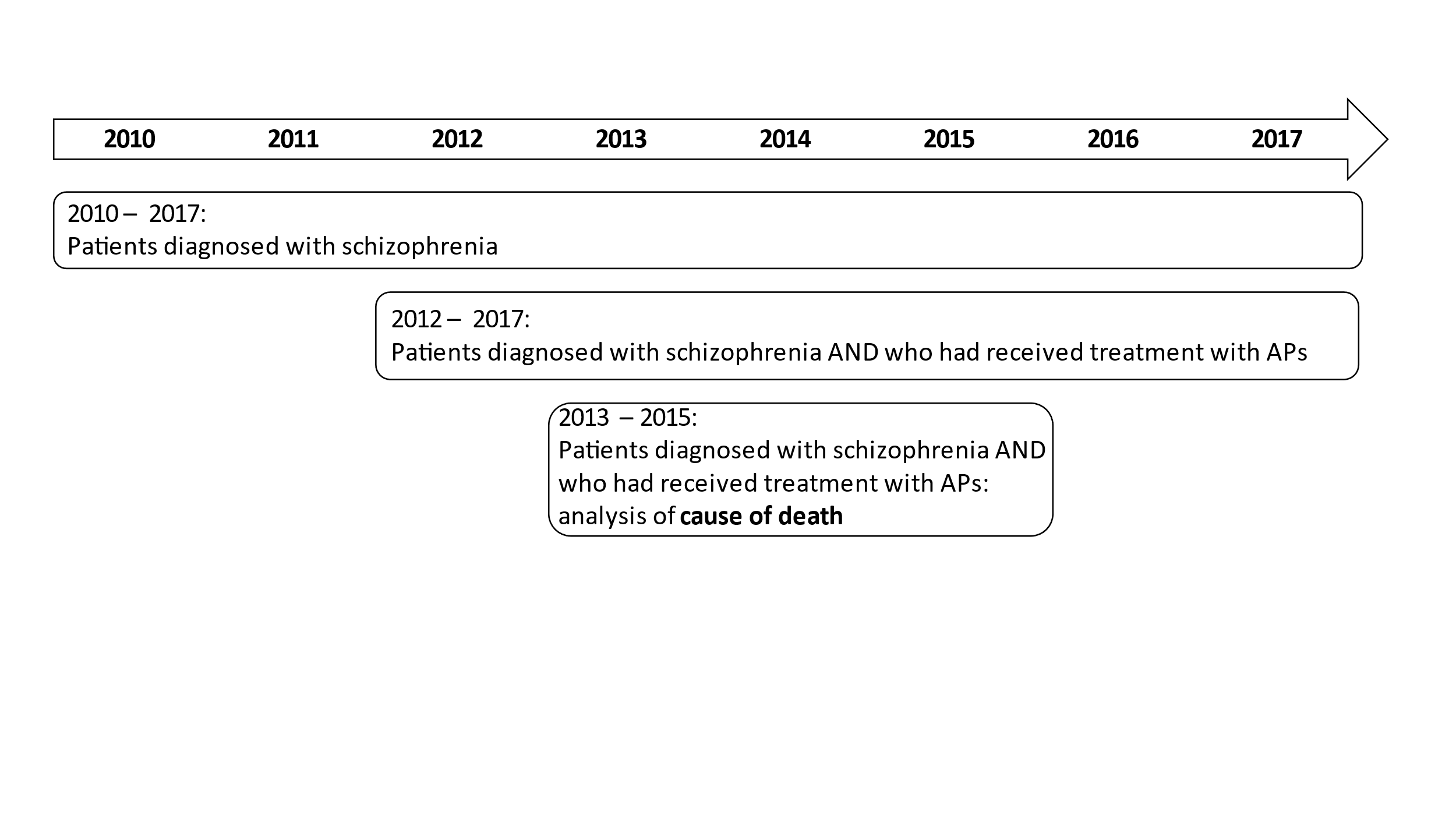


Figure S2. Selection of the study population*


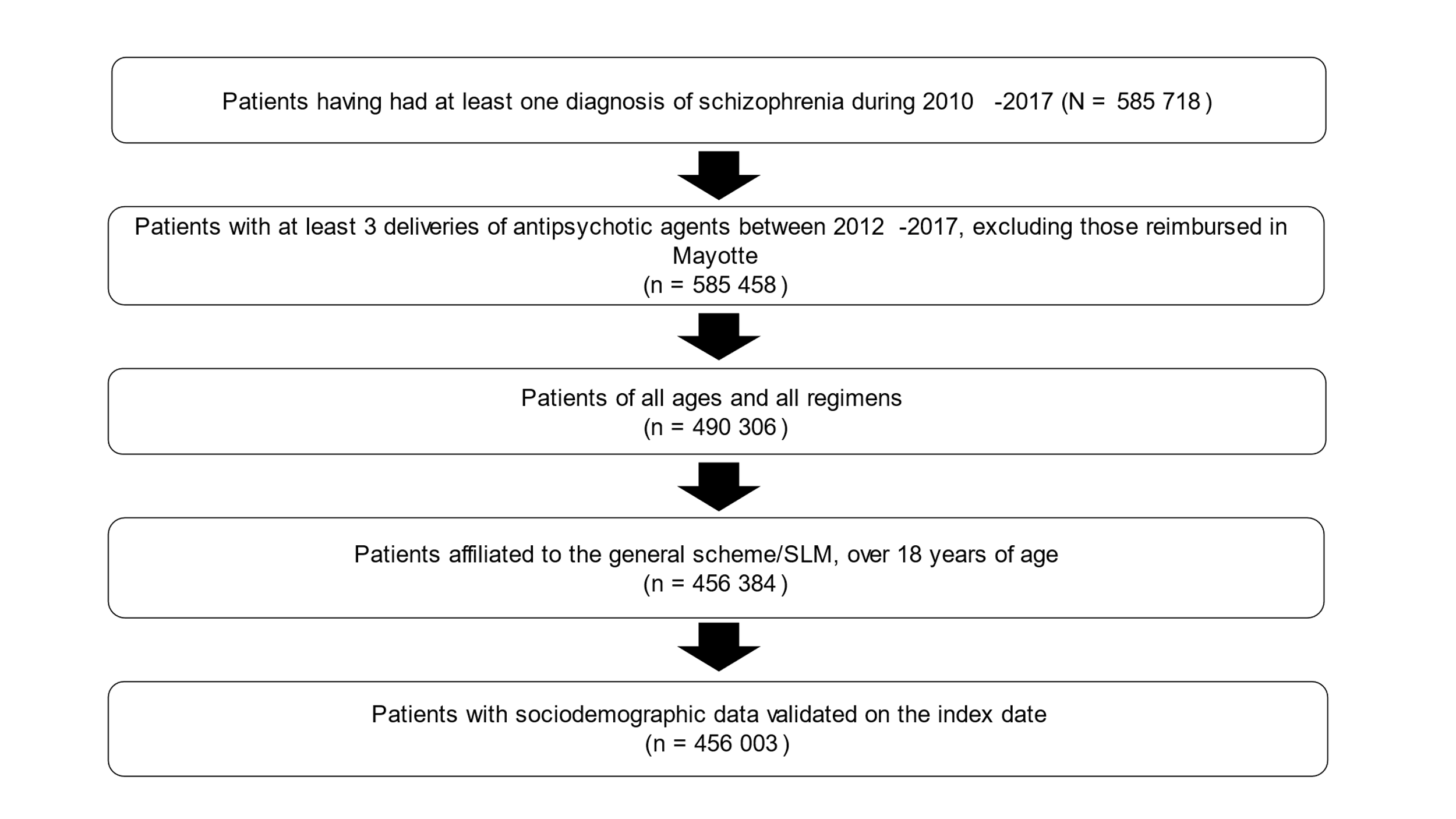


*Patients were excluded from the study if they were under 18 years of age at the index date (defined as the first date of schizophrenia diagnosis or first delivery of an AP) or were insufficiently recognizable as an individual (e.g., fictional ‘pseudoNIR’ [unique personal identification number] individuals, twins). Patients were also excluded if they were not enrolled in the general scheme (régime général [RG]) or a local mutual association (section locale mutualiste [SLM]), or had received treatments reimbursed in Mayotte.
